# P06 Improving antibiotic prescribing practice in a tertiary burns and plastic surgery department

**DOI:** 10.1093/jacamr/dlad066.010

**Published:** 2023-06-14

**Authors:** Cristina Parente, Annie Joseph

**Affiliations:** Nottingham University Hospitals NHS Trust, Nottingham, UK; Nottingham University Hospitals NHS Trust, Nottingham, UK

## Abstract

**Background:**

Antimicrobial resistance is an ever-emerging global public health issue. In the healthcare setting, it is essential to use antimicrobials appropriately and manage infections effectively to help tackle antimicrobial resistance.

**Objectives:**

To review local antibiotic resistance data, update the plastic surgery infection guidelines based on this data, implement interventions to facilitate compliance with the guidelines and re-audit to assess if these interventions have been effective.

**Methods:**

A review was undertaken of the previous 3 months of antibiotic susceptibility results for significant staphylococci and streptococci species isolated from blood cultures and wound swabs, and the microbiology results of all local cases of necrotizing fasciitis over a 5-year period. The plastic surgery infection guidelines were updated to reflect the changes in local antibiotic resistance rates. Following implementation of the new guideline, various interventions were undertaken including provision of departmental teaching sessions, ensuring availability of appropriate antibiotics in the burns and plastics theatres and redesigning the guideline to include all plastic surgery and infection guidelines within the one document (previously multiple specific guidelines). Between each intervention, compliance with the guideline was measured to establish if the intervention had made a difference.

**Results:**

Changes to the guideline included using clindamycin rather than doxycycline for the treatment of cellulitis in penicillin allergic patients, as 44% of Group A Streptococcus isolates identified in wound swabs were resistant to doxycycline. Other changes included the introduction of linezolid if the patient had received a recent or failed course of clindamycin, and the use of cefazolin rather than clindamycin for patients with severe cellulitis with a penicillin allergy, due to the increase in rates of clindamycin resistance. After the three intervention cycles, prescriber compliance with the guideline increased from 50% to 71% demonstrating the need for ongoing intervention and re-evaluation.

**Conclusions:**

Co-amoxiclav and clindamycin were the most commonly prescribed antibiotics in those patients whose antibiotic prescriptions did not comply with the guidelines. The main reason for this is historical usage of these antibiotics within the specialty; this project demonstrates the challenges in changing established practices in individual clinicians. Interventions to ‘make it easy’ for prescribers to follow antibiotic guidelines (for example, the right antibiotic being easily available at the site of prescription), along with targeted education, can be effective but long-standing prescribing habits within specialist areas may be difficult to change.

